# RefEx, a reference gene expression dataset as a web tool for the functional analysis of genes

**DOI:** 10.1038/sdata.2017.105

**Published:** 2017-08-29

**Authors:** Hiromasa Ono, Osamu Ogasawara, Kosaku Okubo, Hidemasa Bono

**Affiliations:** 1 Database Center for Life Science, Joint Support-Center for Data Science Research, Research Organization of Information and Systems, 1111 Yata, Mishima 411-8540, Japan; 2 Center for Information Biology, National Institute of Genetics, Research Organization for Information and Systems, 1111 Yata, Mishima 411-8540, Japan

**Keywords:** Transcriptomics, Gene expression profiling, Genetic databases

## Abstract

Gene expression data are exponentially accumulating; thus, the functional annotation of such sequence data from metadata is urgently required. However, life scientists have difficulty utilizing the available data due to its sheer magnitude and complicated access. We have developed a web tool for browsing reference gene expression pattern of mammalian tissues and cell lines measured using different methods, which should facilitate the reuse of the precious data archived in several public databases. The web tool is called Reference Expression dataset (RefEx), and RefEx allows users to search by the gene name, various types of IDs, chromosomal regions in genetic maps, gene family based on InterPro, gene expression patterns, or biological categories based on Gene Ontology. RefEx also provides information about genes with tissue-specific expression, and the relative gene expression values are shown as choropleth maps on 3D human body images from BodyParts3D. Combined with the newly incorporated Functional Annotation of Mammals (FANTOM) dataset, RefEx provides insight regarding the functional interpretation of unfamiliar genes. RefEx is publicly available at http://refex.dbcls.jp/.

## Introduction

Profiling gene expression of tissues is important for the study of gene function. Clues to gene function can often be obtained by examining when and where a gene is expressed in the tissues and cell lines. The gene expression patterns in various tissues and cell lines obtained by different quantification methods are helpful to properly infer the functions of unfamiliar genes in conjunction with available functional annotation of genes.

Gene expression data are exponentially accumulating with the advancement of gene-expression measurement methods on a genomic scale. The first attempt was to measure expression by counting the number of expressed sequence tags (ESTs) sequenced by traditional Sanger methods in different tissues^1,2^. After the invention of microarray, it was soon used for tissue profiling in the Functional Annotation of Mammals (FANTOM) project^3–5^. A similar effort for Affymetrix GeneChip microarrays was made by the BioGPS group^6^.

In conjunction with advances in RNA sequencing (RNA-seq) technology, the high-throughput identification of transcription start sites became possible using the cap analysis of gene expression (CAGE), which was developed by the RIKEN group^7^. Using this technology, the FANTOM collaboration consortium released terabytes of transcriptome sequencing data from adult and fetal human and mouse tissue primary cell lines that were obtained by the FANTOM5 project^8,9^.

The availability of such data is quite beneficial to biologists who wish to reuse it, but accessing the data remains difficult due to its sheer magnitude and complicated access. Recently, a meta-analysis of RNA-Seq expression data across various species, tissues, and studies was performed^10^; however, the interpretation of such data is difficult. Biologists are often at a loss because of the sheer number of datasets in public databases provided by numerous researchers. From such situations, reference expression datasets are needed for the inference of functions of genes, and a proper web interface for visualizing such data is essential.

In addition, concerted patterns of gene-expression profiles for different quantification methods can strengthen the evidence of these patterns. Also, tissue specific expression can be a key feature to examine the function of genes of interest, and lists of genes with tissue specific expression can help biologists to explore unannotated genes with prominent expression patterns. Thus, the functional annotation of genes from meta-analysis and the interface to access the data with graphical visualization are urgently required.

We have developed a web tool for browsing reference gene expression, which provides access to curated data from several other public databases, with expression levels in forty tissues measured by four well-established gene-expression quantification technologies. The web interface allows users to browse the expression profiles by the gene name, various types of IDs, chromosomal regions in genetic maps, gene family based on InterPro^11^, gene expression patterns, or biological categories based on Gene Ontology^12^. The web interface, named Reference Expression dataset (RefEx), allows users to compare expression profiles by different methods at a glance (http://refex.dbcls.jp/).

## Results

### Integration of publicly available gene expression data

**Figure 1:**
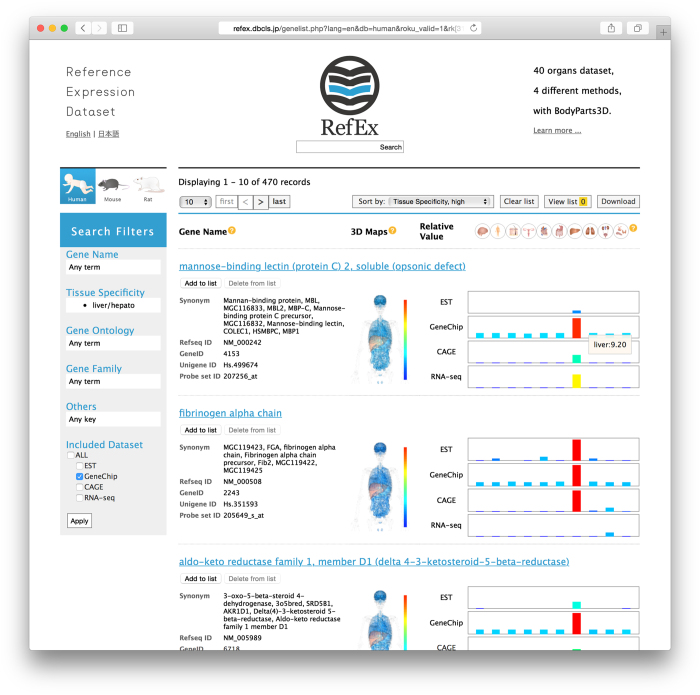
RefEx web interface. This summary page shows the search results for liver-specific genes by clicking the liver icon at the top of the RefEx page (http://refex.dbcls.jp/).

RefEx provides suitable datasets as a reference for gene expression data from 40 normal human, mouse, and rat tissues and cells (http://refex.dbcls.jp). Forty tissues were selected based on the experience gained while constructing the BodyMap database^1,2^. The 40 tissues are classified into 10 groups (i.e., brain, blood, connective, reproductive, muscular, alimentary, liver, lung, urinary, and endo/exocrine) (Table 1). These groupings are mainly used for the abstraction of the gene expression profiles in the summary view (Fig. 1) and the inference of gene functions by the gene expression profiles.

The following four different measurement strategies were used in our collected gene expression data: ESTs, Affymetrix GeneChip, CAGE, and RNA-Seq. These four types of data were linked based on the NCBI gene IDs in the dataset in RefEx. The EST data were clustered by sequence similarity, and the NCBI UniGene^13^ IDs were added to those clusters. The Affymetrix GeneChip data were based on Affymetrix probe IDs, which were originally designed based on the UniGene database. The remaining two methods were based on direct sequencing and were developed after the completion of the human and mouse genome sequencing projects; the data obtained by these methods can be mapped to the reference genomes by the genomic position. Thus, we began using the NCBI Gene IDs, which are currently widely used to integrate other gene IDs, as a standard. Mapping the various gene IDs (UniGene ID, Affymetrix probe ID, and NCBI Gene ID) onto the various genomes was performed using the Biomart REST API (http://www.biomart.org/martservice.html). In addition to the description of the RefEx dataset production in the Methods section, all scripts are available at https://github.com/dbcls/RefEx.

The original data sources for all data records are summarized in Table 2. The processed gene expression data are deposited in figshare (Data Citation 1).

### Visualization with BodyParts3D

The relative gene expression values are shown in RefEx as choropleth maps on 3D human body images from BodyParts3D^14^. BodyParts3D, which was developed by the Database Center for Life Science (DBCLS), is a dictionary-type anatomy database in which anatomical concepts are represented by 3D structural data that specify the corresponding segments on a 3D whole-body model of an adult human male^15^. Foundational Model of Anatomy (FMA) ontology (https://bioportal.bioontology.org/ontologies/FMA) was used to map the gene expression data onto the corresponding tissues. Because drawing the choropleth maps dynamically on a 3D human body is quite labor-intensive, still images were prepared for only the GeneChip data for the whole entries. Figure 1 clearly illustrates that the selected transcript is highly expressed in the liver tissue. This type of visualization can help users to understand the differences in the gene expression patterns among tissues more intuitively. On the right (Fig. 1), the relative expression levels in 40 types of normal tissues that were more precisely classified are displayed.

RefEx has a search filter to extract genes with concerted gene expression profiles. For example, a user can retrieve genes with liver-specific gene expression using the four methods and can quickly obtain such search results (https://youtu.be/Jfo0Uquz15U?t=5m23s).

At the gene level, Troponin T type 2 is a good example of a concerted expression pattern using the four different methods (https://youtu.be/Jfo0Uquz15U?t=2m).

**Figure 2:**
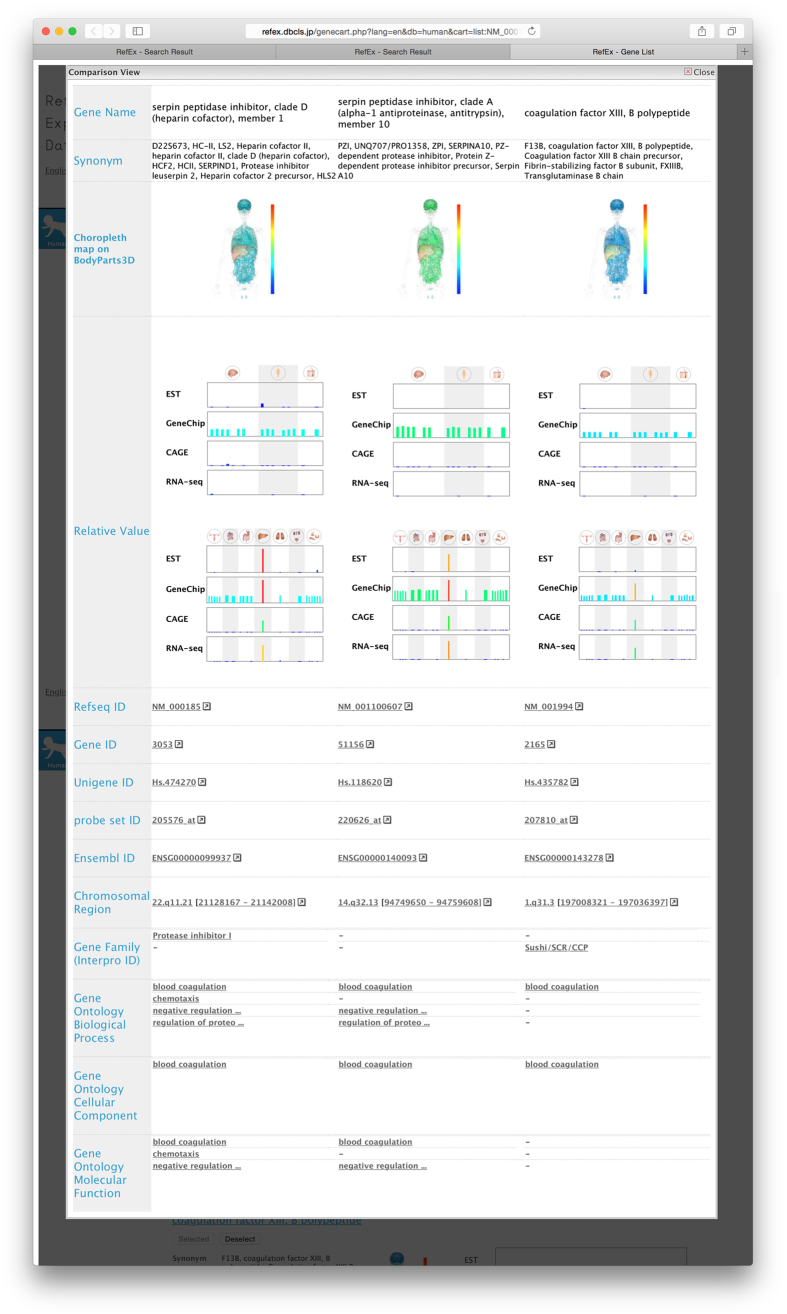
Comparison view. Up to three genes can be compared simultaneously. Users can compare all detailed information in parallel. The expression data and the overlapped annotated terms from Gene Ontology and the InterPro gene family are arranged in the same row.

Users can add up to three genes to their list and compare these genes simultaneously. Users can compare all the detailed information about the genes in that list, including the expression data. This parallel comparison enables users to easily identify the differences among the genes. Overlapped terms, such as the Gene Ontology^12^ and the InterPro gene family^11^ terms, are arranged in the same row (Fig. 2). Therefore, RefEx is also useful as a tool for investigating the relationships of unknown genes found in gene expression analyses.

A RefEx help page, entitled ‘How to use RefEx’ (http://refex.dbcls.jp/help.php?lang=en), provides information about RefEx not only as a text manual but also as a tutorial movie (http://doi.org/10.7875/togotv.2016.068). The movie originated from the contents of TogoTV^16^, which provides tutorial videos for useful databases and web tools in the life sciences, and is available on the TogoTV original website^17^ and YouTube.

### Extraction of genes with tissue-specific expression patterns

RefEx contains unique lists of genes with prominent expression patterns in a specific tissue relative to those in other tissues. The genes with tissue-specific expression patterns are calculated for all tissues using the ROKU method^18^. ROKU was originally developed solely for extracting tissue-specific genes. ROKU ranks the genes according to their overall tissue specificity using Shannon entropy and detects the tissues that are specific to each gene, if any exist, using an outlier detection method. We introduced ROKU in our study because it was superior to conventional entropy-based methods for the detection of genes with expression patterns that are specific only to target tissues. ROKU is implemented as a TCC package^19^ in the R programming language, and we used this package in RefEx. Clicking on the tissue icons on the top of the RefEx page easily retrieves genes with tissue-specific expression patterns.

Users can easily change the order of the genes in the list using a variety of expression values and gene names, while the default gene list order is sorted according to the tissue specificity calculated using the ROKU method. The most prominent usage of this sorting is to utilize the ‘gene2pubmed’ dataset and the manually curated ‘genes to PubMed literature’ relationship dataset provided by NCBI. Furthermore, sorting genes by the number of corresponding literature citations in PubMed enables users to sort according to whether a gene is ‘famous’^20^. This sorting option enables users to discover promising genes with unknown functions.

### Gene expression visualization of the FANTOM5 CAGE data

Recently, we incorporated the CAGE data from the FANTOM5 project^9^ into RefEx. The FANTOM5 project is a broad atlas of gene expression in humans and mice. The most important benefit of the FANTOM5 CAGE data is that the search targets are much more abundant. The original version of RefEx only had 40 tissue search targets. However, it is now possible to search more than five hundred human samples, encompassing cell lines, primary cells, and adult and fetal tissues. RefEx also enables users to browse high-resolution gene expression data from approximately eight hundred samples (human plus mouse). A relevant example of this additional data is the gene expression profile of induced pluripotent stem cells (iPS cells). Weak but biologically meaningful gene expression patterns can be detected via the RefEx interface.

**Figure 3:**
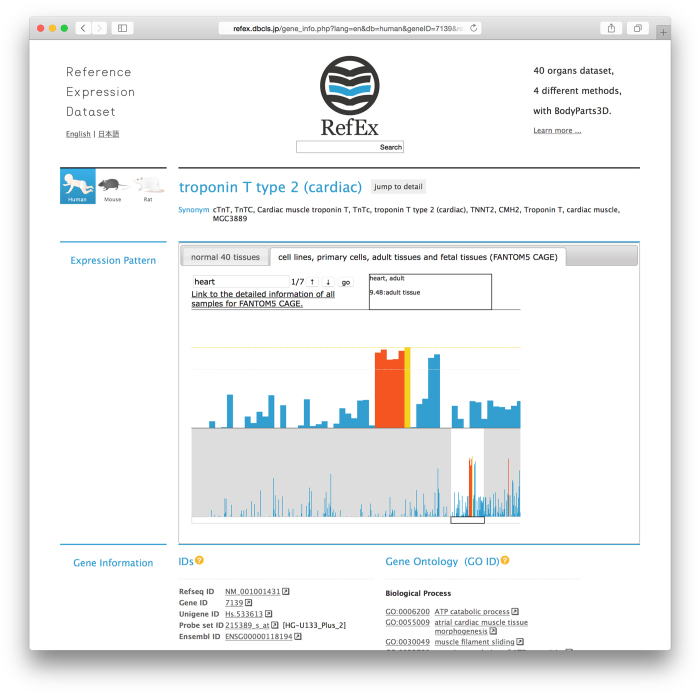
FANTOM5/CAGE data viewer. By clicking on the tab on the right-hand side, users can switch to a FANTOM5 CAGE data viewer. This viewer shows the expression patterns of all samples from the FANTOM5 CAGE data in the lower portion of the screen and displays an enlarged view of a specific area in the upper portion of the screen.

By clicking the tab on the right-hand side, users can switch to a FANTOM5 CAGE data viewer (Fig. 3). This viewer shows the expression patterns of all samples in the lower portion of the screen and displays an enlarged view of a specific area in the upper portion of the screen. Because this is a representation of the expression profile in humans, 556 samples are shown in a bar chart in the lower portion of the screen. Therefore, a user can observe an overview of expression patterns in all the samples. The area displayed in the enlarged box can be moved freely by dragging. When a user enters a keyword into the search window of the viewer, the sample name containing that keyword is highlighted. The complete FANTOM5 CAGE data sample names list is accessible in the figshare public data repository (Data Citation 2 for human samples and Data Citation 3 for mouse samples). The FANTOM5 CAGE data correspond to the tissue classification in the original RefEx and are linked to the original FANTOM5 data. The expression values of the samples obtained in the FANTOM5 project are averaged and listed in RefEx.

## Discussion

RefEx is now accessible and allows for an interactive analysis of gene expression patterns on the web via the latest version of web browsers such as Firefox, Safari, and Chrome. RefEx has three main applications.

First, users can examine the expression profiles of unfamiliar genes in normal body tissues, cells, and cell lines based on actual measurement data rather than only from a description in a journal article. Second, a search for tissue-specific genes can be performed simply by clicking on the appropriate tissue icon at the top of the RefEx page. Third, users can compare differences in gene expression levels related to the use of different experimental methods.

RefEx is freely available not only to academic users but also to for-profit users under a Creative Commons Attribution 4.0 International License. Users might prefer to download the data to share and analyze locally with other software. Thus, a user can download a concatenated version of all the data at the RefEx website, including the sequences, the functional annotation of the cDNA clones, and the log-transformed ratios of the gene expression, in a tab-delimited text format. The RefEx data download page (http://refex.dbcls.jp/download.php?lang=en) is also available at github (https://github.com/dbcls/RefEx/) and figshare (Data Citation 1).

RefEx has recently contributed to several medical research fields. One prominent example is in the field of cancer research. The mRNA expression levels of IDH3α and VEGF-A were used and visualized as main indicators in 10 major groups of normal tissues^21^ in conjunction with the calculated prognostic values by the PrognoScan database^22^. Another example is a study of murine colon proteomes in which colon-specific genes in the mouse version of RefEx were compared to a list of genes from murine colon proteomes that was generated by the researchers’ own results^23^. A similar example was a study of liver-specific genes to investigate biomarkers indicating liver injury in humans. The gene expression profiles of ALB, APOH, GC, and AMBP were used to confirm the liver-specific expression^24^. The gene expression profile of noncardiac MRLC (myosin, light chain 12A, NM_006471) in RefEx was used to confirm the conclusions because noncardiac MRLC was expressed in the heart at the same level as that in the skeletal muscle while it was annotated as ‘non-cardiac’^25^. Furthermore, in a review article, RefEx was used to list the gene expression profiles of all genes previously reported to cause deafness^26^. The data retrieved from RefEx strengthened the authors’ hypotheses without the need for further wet-lab experiments in all cases.

There are already several projects to organize reference expression dataset. The European Bioinformatics Institute (EBI) maintains the Expression Atlas^27^, which provides information regarding gene expression patterns under different biological conditions based on data archived in the public gene expression database ArrayExpress^28^. Swiss Institute of Bioinformatics maintains Bgee (a database for gene expression evolution). Bgee is a database that constructs a reference dataset for gene expression by integrating and comparing heterogeneous transcriptome data among species^29^. Users can easily determine where and when their genes of interest are expressed or not expressed in various species. ReCount also contains analysis-ready RNA-Seq gene count datasets^30^. In addition, a similar tool named OMics Compendia Commons (OMiCC), which is freely available and aims to assist biologists with limited bioinformatics training, was recently reported^31^. OMiCC enables the broader biomedical research community to generate and test hypotheses through the reuse and (meta-)analysis of existing datasets. Reference expression datasets are needed for the inference of the functions of genes, and a convenient web interface for visualizing such data is essential.

Currently, our transcripts, which are based on RefSeq mRNA records, are used to integrate different types of measurement methods for gene expression. However, according to a high-throughput sequencing data analysis, over 90% of human genes undergo alternative splicing^32^, and many of these are not yet included in RefSeq. To address this limitation, the definitions of the transcripts need to be redefined to include noncoding RNA in tight collaboration with the FANTOM project. In the upcoming version of RefEx, we also plan to use personalized gene expression data from the Genotype-Tissue Expression database (GTEx)^33^. We currently have direct web links to the corresponding GTEx gene report pages in the human version of RefEx.

As a member of the integrated database project in Japan, the Resource Description Framework (RDF) version of RefEx resides at the National Bioscience Database Center (NBDC) RDF portal, and the RefEx dataset is ready for use at the NBDC RDF Portal (http://integbio.jp/rdf/). The Expression Atlas^27^ also provides the dataset in RDF format as a beta version (https://www.ebi.ac.uk/rdf/services/atlas/).

## Methods

### Data collection

The data in RefEx were manually collected by RefEx curators from public databases, including the International Nucleotide Sequence Database (INSD, consisting of GenBank/DDBJ/ENA)^34^, RNA-Seq database in the Sequence Read Archive (SRA)^35^, and the NCBI Gene Expression Omnibus (GEO)^36^. The raw data from the public databases were re-organized and compared against each other. Four types of data were linked based on the NCBI gene IDs, while the EST data were based on the Unigene IDs, and the GeneChip data were based on the Probe set IDs. Detailed information regarding our four data extraction methods are described below. All scripts used to produce the data and additional descriptions are available on the github site at https://github.com/dbcls/RefEx.

### EST

The EST data were originally obtained from the EST division of the INSD^34^. The number of ESTs was counted by source organ based on the BodyMap method^1^ according to the cDNA annotation of each EST entry. The EST data in RefEx originated from the BodyMap-Xs database, which contains previously compiled gene expression data from the INSD EST division for reuse^2^.

After our cDNA library analysis, we obtained EST gene expression data for the 40 normal tissues that are stored in the BodyMap-Xs database, and these data are listed in Table 1. For visualization purposes, we grouped the data into 10 subsets (i.e., brain, blood, connective, reproductive, muscular, alimentary, liver, lung, urinary, and endo/exocrine). This categorization of the organs was also applied to the gene expression data that were obtained by the other methods.

### GeneChip

The GeneChip data were previously measured by Affymetrix microarrays (GeneChip) and calculated based on a typical microarray data analysis method^6^. We extracted the microarray data deposited in the NCBI GEO database for our reference dataset (tissue-specific patterns of mRNA expression) by selecting the GEO Series accession numbers GSE7307 for human, GSE10246 for mouse, and GSE952 for rat. The expression values of the genes were calculated from the original CEL files after robust multi-array averaging (RMA) normalization^37^ by the affy package^38^ in R (ver.3.0.3)/BioConductor^39^ (ver.2.12).

### CAGE

CAGE is a technique that produces a snapshot of the 5′ end of the mRNA population in a biological sample^9^. The CAGE data collected in the RIKEN FANTOM5 project were counted by source organ based on the original data, the FANTOM5 CAGE peak expression, and the annotation tables. The CAGE tag counts were mapped onto the reference genome sequences (hg19 for human and mm9 for mouse) and reflect the intensity of the gene expression of the corresponding transcripts. The tag counts are normalized by tag per million (TPM). In addition to the 40 tissues listed in Table 1, the FANTOM5 project collected hundreds of samples from human and mouse cell lines, primary cells, and adult and fetal tissues. The complete list of these samples is available in figshare (Data Citation 2 for human samples and Data Citation 3 for mouse samples).

### RNA-Seq

We extracted the normal tissue transcriptome sequence data from the SRA^35^. The corresponding expression level and location data originated from the Illumina BodyMap 2 project for human and mouse transcriptomes (Table 2). These data were processed using a typical RNA-Seq data analysis pipeline with TopHat (ver.2.0.7) and Cufflinks (ver.2.0.2), and the transcript abundances were calculated and normalized to fragments per kilobase of transcript per million reads (FPKM)^40,41^. The reference genome sequence versions were human hg19 and mouse mm9.

All data described above are archived under the figshare Project ‘Data Archive for RefEx’ (Data Citation 1).

## Technical Validation

### Authenticity of the data used in RefEx

Biological replicates of gene expression experiments were averaged to represent the gene expression value. The EST data were directly imported from the BodyMapXS database^2^. The frequency of the EST counts was used as a measure of the gene expression intensity. The validation of its quality is difficult because BodyMapXS is currently closed, but it has been used for identifying genes as a source for the ESTs.

Regarding the GeneChip data, we validated the expression of the genes by the intensity of the microarray spots. The intensity of these spots can be searched via the NCBI GEO2R interface (‘Value distribution’ tab). The following URLs are direct links to the corresponding data for the distribution of the gene expression intensities, and we confirmed that all the microarray data had the proper range of intensities.

- human: https://www.ncbi.nlm.nih.gov/geo/geo2r/?acc=GSE7307
- mouse: https://www.ncbi.nlm.nih.gov/geo/geo2r/?acc=GSE10246
- rat: https://www.ncbi.nlm.nih.gov/geo/tools/profileGraph.cgi?ID=GDS589

We used the CAGE data from the RIKEN FANTOM5 project. The expression values were already converted to TPM and were provided on the original site. In the FANTOM5 project, the appropriate filtering methods were based on the quality of the raw data and were applied as shown on the FANTOM5 website at http://fantom.gsc.riken.jp/5/sstar/Protocols.

Thus, the quality of the CAGE data was technically sound for reuse in RefEx.

Regarding the RNA-Seq data used (Data Citation 4), single-read and paired-end sequencing were attempted in humans. The gene expression intensities were calculated by the pipeline described in the Method section. Finally, these intensities were averaged to represent the gene expression values in humans. Regarding the mouse RNA-Seq data used (Data Citation 5), triplicate single-read sequencing was available. Similar to the data processing in humans described above, these data triplicates were averaged to represent the gene expression value in mouse. The sequence quality for all datasets calculated by the fastqc program is available on the DBCLS SRA web site. For example, the sequence quality data used in the human dataset is accessible via the URL http://sra.dbcls.jp/search/view/ERP000546. The sequence quality was determined to be above 30 in the human dataset.

Thus, we conclude that the EST and microarray data were not invalid data and that the data quality of the sequencing assays (CAGE and RNA-Seq) were good enough for reuse in RefEx.

### Gene expression patterns obtained by different methods

Ideally, gene expression patterns should be the same in data obtained by different methods. Troponin T type 2 is a good example of concerted expression pattern obtained using the four different methods.

http://refex.dbcls.jp/gene_info.php?lang=en&db=human&geneID=7139&refseq=NM_001001431&unigene=Hs.533613&probe=215389_s_at

However, gene expression patterns can be different using different methods because of experimental bias. To validate the gene expression patterns obtained using different methods, genes with human liver-specific expression were compared using the tissue-specific dataset in RefEx.

In GeneID, 232 unique genes were determined to have liver-specific expression based on the GeneChip data, while 472 unique genes were determined to have liver-specific expression based on the RNA-Seq data.

**Figure 4:**
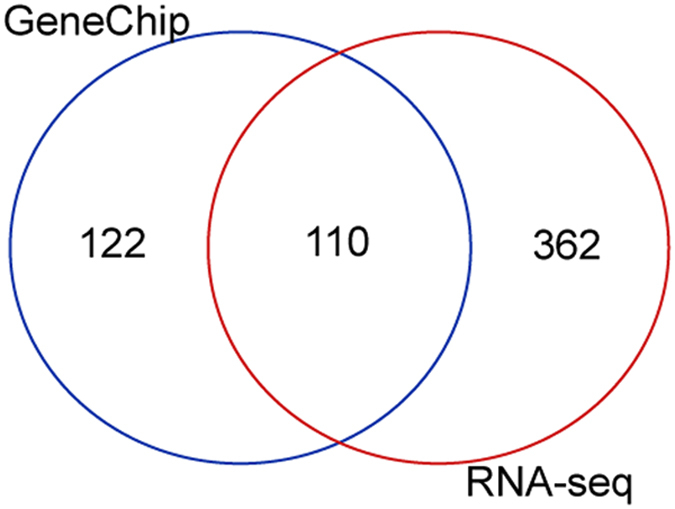
Comparison of human liver-specific genes obtained using different quantification methods for gene expression. A Venn diagram of the human liver-specific genes in the GeneChip data and the RNA-Seq data. The Venn diagram was generated using the ‘Draw Venn Diagram’ website at http://bioinformatics.psb.ugent.be/webtools/Venn/.

A Venn diagram represents the differences between these two datasets and shows that 110 genes were shared in the two datasets (Fig. 4).

Because GeneChip measures the expression of genes only on the microarray, while RNA-Seq analyzes the detailed gene expression by deep sequencing, approximately twice the number of genes were determined to be liver-specific in the RNA-Seq dataset than in the GeneChip dataset. Considering the heterogeneity of the gene expression data obtained using the different methods, users can benefit from the knowledge that half of the genes in GeneChip were determined to have gene expression.

Thus, we conclude that the overall reproducibility of the different types of gene expression data-sets is good enough for reuse in RefEx.

### Construction of the RefEx website

The RefEx web server operates on CentOS (Linux) and is implemented on an Apache web server with PHP in conjunction with a MySQL database. Access to the RefEx website is optimized for the latest versions of Safari, Firefox, and Google Chrome. Additional software is not needed. All data on our web server are stored in flat file format (tab-delimited text) and are loaded in our MySQL database for visualization on the web.

## Additional Information

**How to cite this article:** Ono, H. *et al.* RefEx, a reference gene expression dataset as a web tool for the functional analysis of genes. *Sci. Data* 4:170105 doi: 10.1038/sdata.2017.105 (2017).

**Publisher’s note:** Springer Nature remains neutral with regard to jurisdictional claims in published maps and institutional affiliations.

## Figures and Tables

**Table 1:** Forty mammalian RefEx tissues grouped into 10 categories.

Brain	Cerebrum	Cerebellum	Brain stem	Corpus callosum/glia	Pineal gland	Peripheral nerve	Spine	Retina	Eye
Blood	Artery/Aorta	Vein	Lymph node	Peripheral blood	Spleen	Thymus	Bone marrow		
Connective	Adipose	Bone	Skin						
Reproductive	Uterus	Placenta	Prostate	Ovary	Testis				
Muscular	Heart	Muscle							
Alimentary	Esophagus	Stomach	Intestine	Colon					
Liver	Liver/Hepato								
Lung	Lung								
Urinary	Bladder	Kidney							
Endo/Exocrine	Pituitary	Thyroid/Parathyroid	Adrenal gland	Pancreas	Breast	Salivary			

**Table 2:** Original data source.

	**Human**	**Mouse**	**Rat**
EST	INSD	INSD	INSD
GeneChip	GSE7307	GSE10246	GSE952
CAGE	PRJDB3010	PRJDB1100	Not Available
RNA-Seq	PRJEB2445	PRJNA30467	Not Available
As described in the main text, the EST data originated from the EST division of the combined GenBank/DDBJ/ENA databases (INSD)^34^. The primary GeneChip data source is the NCBI Gene Expression Omnibus (GEO) database^36^. The SRA^35^ provided the CAGE and RNA-Seq data, and the corresponding IDs in the BioProject database are described in the table.			
